# Digital Technologies for Monitoring and Improving Treatment Adherence in Children and Adolescents With Asthma: Scoping Review of Randomized Controlled Trials

**DOI:** 10.2196/27999

**Published:** 2021-09-17

**Authors:** Madison Milne-Ives, Ching Lam, Edward Meinert

**Affiliations:** 1 Centre for Health Technology University of Plymouth Plymouth United Kingdom; 2 Institute of Biomedical Engineering Department of Engineering Science University of Oxford Oxford United Kingdom; 3 Department of Primary Care and Public Health Imperial College London London United Kingdom; 4 Harvard T.H. Chan School of Public Health Harvard University Boston, MA United States

**Keywords:** asthma, disease management, child, adolescent, telemedicine

## Abstract

**Background:**

Inadequate pediatric asthma care has resulted in potentially avoidable unplanned hospital admissions and morbidity. A wide variety of digital technologies have been developed to monitor and support treatment adherence in children and adolescents with asthma. However, existing reviews need to be updated and expanded to provide an overview of the current state of research on these technologies and how they are being integrated into existing health care services and care pathways.

**Objective:**

This study aims to provide an overview of the current research landscape and knowledge gaps regarding the use of digital technologies to support the care of children and adolescents with asthma.

**Methods:**

This study was structured according to the PRISMA-ScR (Preferred Reporting Items for Systematic Reviews and Meta-Analyses extension for Scoping Reviews) and Population, Intervention, Comparator, Outcome, and Study frameworks. Five databases (PubMed, the Cochrane Central Register of Controlled Trials, Web of Science, Embase, and PsycINFO) were systematically searched for studies published in English from 2014 onward. Two reviewers independently screened the references and selected studies for inclusion based on the eligibility criteria. Data were systematically extracted per research question, which were synthesized in a descriptive analysis.

**Results:**

A wide variety of study characteristics, including the number and age of participants, study duration, and type of digital intervention, were identified. There was mixed evidence for the effectiveness of the interventions. Of the 10 studies that evaluated treatment adherence, 7 (70%) found improvements, but the evidence was inconsistent for asthma control (6/9, 67% of studies reported improvement or maintenance, but only 1 was significantly different between groups) and health outcome variables (5/9, 56% of studies found no evidence of effectiveness). The 6 studies that examined patient perceptions and assessments of acceptability and usability generally had positive findings.

**Conclusions:**

A wide range of digital interventions are being developed and evaluated to support the monitoring and treatment adherence of children and adolescents with asthma. Meta-analyses are inhibited by the use of samples with a variety of overlapping age ranges; a theoretical framework for evaluating specific age groups would aid comparison between studies. Most studies found significant evidence for improved adherence to treatment or medications, but there was mixed evidence of the impact of the digital interventions on asthma control and other health outcomes. There are gaps in the literature relating to cost-effectiveness and integration with existing clinical care pathways. This study will be necessary to determine which digital interventions for children and young people with asthma are worth supporting and adopting in the clinical care pathways.

## Introduction

### Background

Globally, asthma is the most common chronic illness affecting children [1-3] and can have serious health consequences. It is one of the key causes of urgent hospital admissions and morbidity in children [3,4]. This is a particularly urgent problem in the United Kingdom. Out of all the Organization for Economic Co-operation and Development countries, the United Kingdom has the third highest risk of death because of pediatric asthma [3,4]. Although specific data are not available for many countries, asthma has high costs worldwide [5]. The variation in mortality across countries suggests that many of the negative outcomes of childhood asthma, for patients and health care systems, are potentially avoidable [6]. Effective management programs are likely to be a cost-effective means of improving asthma control and reducing the economic burden across countries by enabling early and preventive measures to be taken [5].

A growing number of digital technologies are being developed to help the self-management of people with asthma [7-9]. Broadly, digital technologies are electronic systems that can collect, analyze, and share data, and common examples include mobile or web apps, smart devices, and other phone or internet-based interventions. Some evidence suggests that digital interventions can help support asthma health management, particularly by improving medication adherence [10,11]. However, other results, particularly in terms of effectiveness (depending on the outcome examined) [9] and app quality [8], are mixed. Research has also identified limitations in the studies examining these interventions, including inadequate descriptions of digital interventions, a lack of economic analyses, and small sample sizes [10,12].

For digital interventions to be effective, people need to be willing to use them. Although digital interventions have been shown to be generally acceptable to a wider population [11], special consideration is needed when evaluating digital interventions for children and young people. Adolescents are a particularly challenging group to treat, and poor health literacy and self-management skills can affect their treatment adherence and health outcomes [2]. Attitudes toward electronic monitoring devices were found to be mixed in adolescents, depending on how they perceived the intervention [13]. Among those who viewed asthma as a serious threat, the monitoring device was viewed as reassuring. However, many adolescents were suspicious of the device, reporting concerns that it would get them in trouble if they did not adhere properly to their medication and beliefs that their health care providers did not trust them to take the medication [13]. This demonstrates the need to examine digital interventions tailored specifically for children and young people, as their needs and responses to the interventions may not be the same as the general population.

### Rationale

Although several systematic reviews have examined various topics related to digital interventions for asthma management, there is a need for a comprehensive overview of the evidence being gathered to assess the effectiveness of various types of digital interventions for children and young people with asthma. No previous reviews have been identified that are specific to this population but are broad in terms of the digital interventions examined.

Of the systematic reviews that have focused specifically on children and young people, the scope was limited with respect to either outcome (eg, a focus on treatment adherence [14]) or type of digital technology (eg, only mobile apps [10] or smart devices [15]). One review provided a comprehensive assessment of other systematic reviews [12]. However, this review was published in 2014; given the rapid evolution of digital technology [16], the state of the field has changed since the review was conducted. For instance, electronic inhaler monitoring is a relatively new development [17,18], with smart inhalers only recently becoming commercially available [19]. Another review analyzed studies of children with a wide range of outcomes—adherence, health outcomes, and user perceptions—but only searched PubMed and Embase databases for the study, which raises the concern that some relevant studies might have been missed [9]. To determine if any relevant reviews were in progress, PROSPERO was searched using several combinations of keywords (*asthma* AND *child* OR *paediatric* OR *pediatric* AND *digital* OR *technology* OR *mHealth* OR *eHealth*). These searches identified one relevant registration: a review that was planned, but not executed, by academics associated with the current research team [20].

No reviews were found that examined how the technologies are integrated into current clinical care pathways for children and adolescents with asthma. This is an important area to examine because digital technologies can provide health care professionals with a large body of information that enables them to personalize asthma care plans and focus on preventive measures [21]. A small study by American physicians identified a mix of perceived benefits, barriers, and concerns about integrating digital technologies in asthma care for adolescents [22]. Further research is needed into how digital interventions are currently integrated with health care services [21], to inform the development of integrated clinical care pathways. An overview of the different types of digital technologies and the different ways they are being integrated with health care systems will help inform the development of effective, technologically enhanced care pathways for children with asthma.

### Objectives and Research Questions

The primary objectives of the scoping review are to assess and summarize the current state of the literature on digitally enhanced asthma care for young people and identify any gaps [23]. Three research questions were developed to focus on the review:

1. How are randomized controlled trials (RCTs) of technologically supported asthma pathways being conducted?
2. What is known about the effectiveness of digital technologies in supporting treatment adherence and remote symptom monitoring in children and adolescents?
3. How are studies examining the integration of digital technology into clinical care pathways for pediatric asthma?

## Methods

### Overview

The review was structured following the Preferred Reporting Items for Systematic Reviews and Meta-Analyses Extension for Scoping Reviews (PRISMA-ScR; Multimedia Appendix 1) [24], and the search strategy was developed using the Population, Intervention, Comparator, Outcome, and Study framework (Textbox 1). No protocol was registered or published for this review. A preliminary review of the literature was conducted to extract Medical Subject Headings (MeSH) terms and keywords for the search. The search was performed in five databases (PubMed, the Cochrane Central Register of Controlled Trials [CENTRAL], Web of Science, Embase, and PsycINFO) using the University of Plymouth’s search tool Primo, with slightly adjusted search terms to fit the specific structure of each database. The search terms were grouped into four themes joined in this structure: asthma (MeSH OR Keywords) AND asthma management (MeSH OR Keywords) AND children (MeSH OR Keywords) AND digital technology (MeSH OR Keywords). Multimedia Appendix 2 lists a complete record of the specific search terms and strings used for each database and the number of references returned. The database searches were completed on December 30, 2020, except for the CENTRAL database, which was searched on December 31, 2020.

The inclusion and exclusion criteria are shown in Textbox 2.

**The Population, Intervention, Comparator, Outcome, and Study framework.**

**Population**

- Children and young people under 18 years of age with asthma.

**Intervention**

- Any digital health technology aiming to support monitoring or treatment adherence of children and adolescents with asthma.

**Comparator**

- No comparator is required.

**Outcome**

- The primary outcome was the evidence for the digital interventions at improving monitoring or treatment adherence. Secondary outcomes included how the research was conducted, evidence for improved health outcomes, cost-effectiveness, and integration of the technology with health care systems.

**Study types**

- Randomized controlled trials that evaluate at least one digital technology to support the care of children with asthma.

**Inclusion and exclusion criteria of the study.**

**Inclusion criteria**

- The review included studies evaluating digital technologies that aim to support the monitoring or treatment adherence of children and adolescents aged below 18 years with asthma.
- Digital technologies included, but were not limited to, mobile or web apps, smart devices, and other phone or internet-based interventions.
- Initially, randomized controlled trials, quantitative, qualitative, cohort, and case study types were eligible for inclusion.
- Given the number of studies identified, only randomized controlled trials were included in the review.
- As the scope of the review was focused on assessing evidence of the effectiveness of digital technologies for asthma monitoring and treatment adherence, it was appropriate to limit the included studies to randomized controlled trials that can evaluate effectiveness.

**Exclusion criteria**

- Studies with adult participants were excluded during screening, and studies that only included adults were excluded during the full-text review.
- Studies published before 2014 were excluded to limit the review to the current technologies.
- Studies that merely described an intervention without evaluation were excluded.
- Studies published in languages other than English were also excluded, as the review team did not have the necessary resources to assess them.

### Screening and Article Selection

References were exported to the citation management software EndNote X9 (Clarivate Analytics) for storage and duplicate removal. Owing to the returning of the large number of references, an initial screening was conducted by inputting keywords relating to the inclusion and exclusion criteria into the EndNote X9 search function. This was done in several stages, with each subsequent screening being conducted on the subset of studies retrieved in the previous stage. For example, keywords relating to digital technologies were searched for in any field, and studies that did not contain at least one of those keywords were excluded. Subsequent searches used keywords to exclude studies that used terms unrelated to the topic (eg, cancer, diabetes, and enzyme). Multimedia Appendix 3 contains a full description of the searches conducted. Searches of keywords to exclude were based on common features of irrelevant studies that were identified in a manual search. The remaining titles and abstracts were screened by 2 reviewers (MMI and CL) independently (with articles excluded with reasons), and the final eligibility was determined by full-text reviews of the remaining references. Any discrepancies between the reviewers were discussed until a consensus was reached.

### Data Extraction

Outcomes were extracted by a reviewer (MMI) into a table structured according to the 3 research questions (Multimedia Appendix 4) and verified by a second reviewer (CL). Key outcomes were predetermined based on a preliminary review of the literature; however, because of the expected variety of reported outcomes, relevant outcomes that were not prespecified in the Population, Intervention, Comparator, Outcome, and Study framework or data extraction tables were also considered for inclusion in the final review (Textbox 3).

**Article information and data extraction.**

**General study information**

- Year of publication
- Sample size
- Age of participants

**Digital technology**

- Type of digital technology
- Health care setting used in

**Evaluation**

- Effect of technology on behavioral outcomes (eg, medication adherence and symptom monitoring and reporting)
- Effect of technology on health outcomes
- Cost-effectiveness of the intervention
- Integration of the technology with a health system or care pathway
- Participant perceptions
- Acceptability
- Usability
- Other key performance indicators reported

### Data Analysis and Synthesis

The data extracted from the studies about the key outcomes listed in Textbox 3 were assessed using descriptive analysis and summarized to provide an overview of the state of the literature. For outcomes related to effectiveness, the number of studies that found strong evidence of effectiveness was compared with the number of studies that assessed the outcome to provide a synthesis of the state of the evidence for that outcome. Implications of the findings were examined in the discussion.

## Results

### Included Studies

**Figure 1:**
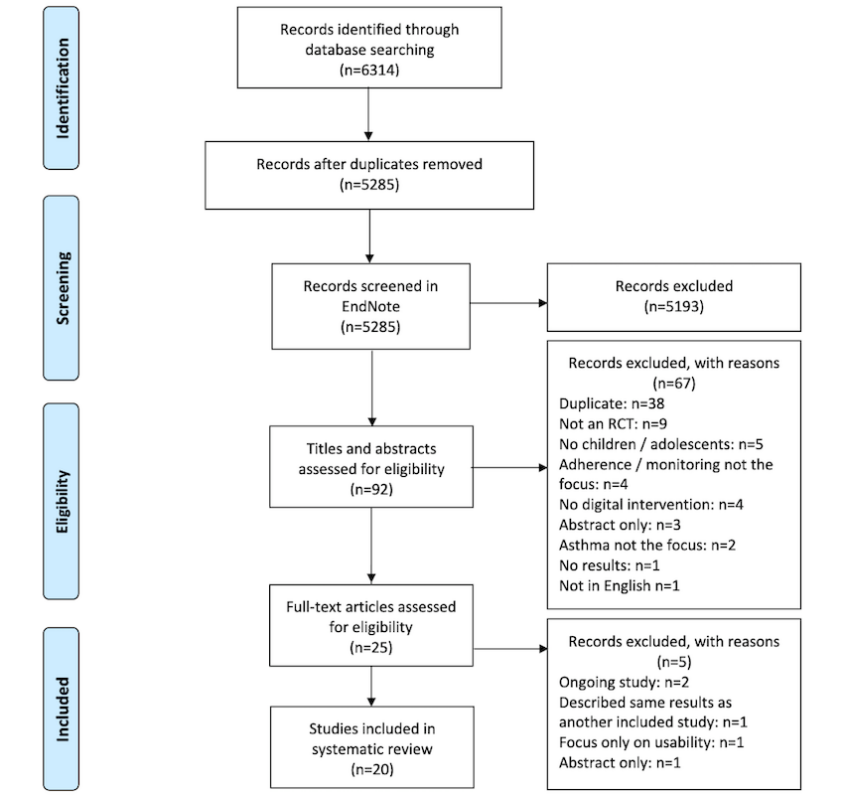
PRISMA (Preferred Reporting Items for Systematic Reviews and Meta-Analyses) flow diagram. RCT: randomized controlled trial.

A total of 6314 articles were retrieved from the search of the 5 databases (Multimedia Appendix 2). A total of 1029 duplicates were removed by the EndNote X9 software, and a further 5193 were screened using keyword searches in EndNote (Multimedia Appendix 3). The titles and abstracts of 92 studies were screened and articles were excluded with reasons. Of these articles, 25 were selected for full-text review, and 20 were selected for inclusion in the review. Of the total references, 6 referred to one study and were either conference abstracts or did not include the final results of the RCT. The paper with published results of the RCT of that study was identified and included [25]. Three references that only provided abstracts subsequently had full texts identified; these full texts were cited and used for data extraction and analysis. The reasons for exclusion in the full-text review stage are shown in Figure 1.

### Study Characteristics

All the studies included in the review were RCTs and limited to those that included monitoring or adherence functions and aims. Despite these restrictions to the scope of the review, the included studies had a wide variety of study durations, sample sizes, age ranges, and types of digital intervention (Table 1).

**Table 1:** Summary of characteristics of 20 identified studies (N=20).

Study	Year	Study duration	Number of participants	Age of participants (years)	Type of digital intervention
Beerthuizen et al [26]	2016	12 months	272 (280 enrolled)	4-18	Web-based monitoring
Bender et al [27]	2015	24 months	1187	3-12	Speech recognition automated telephone program
Britto et al [28]	2017	6 months	64	12-22	Text message reminders
Chan et al [29,30]	2015, 2017	6 months	220	6-15	Inhaler EMD^a^ with audiovisual reminders
Goossens et al [31]	2014	12 months	209	4-11	EMD with text messages
Johnson et al [32]	2016	3 weeks	98	12-17	Website and text-based reminder system (MyMediHealth)
Kosse et al [25,33,34]	2019	6 months	234 (66 pharmacies)	12-18	App (ADAPT^b^)
Merchant et al [35]	2014	100 days	368 (490 enrolled)	5-80	EMD with feedback and educational content
Morton et al [36]	2017	12 months	77 (90 enrolled)	6-16	EMD with alarms and feedback
Perry et al [37]	2018	3 months	393	7-14	School-based educational telemedicine intervention
Real et al [38]	2019	4 months	40	4-11	Gamified app (CHANGE Asthma)
Reece et al [39]	2017	4 months	48	13-60	App (AsthmaWin)
Shields et al [40]	2017	12 weeks	22	2-16	MDOT^c^
Simoneau et al [41]	2019	6 months	43	8-17	EMD with reminders
van den Wijngaart et al [42]	2017	16 months	210	6-16	Web-based monitoring (Virtual Asthma Clinic)
Vasbinder et al [43]	2016	12 months	209	4-11	EMD with text messages
Voorend-van Bergen et al [44]	2015	12 months	268 (280 enrolled)	4-18	Web-based monitoring

^a^EMD: electronic monitoring device.

^b^ADAPT: ADolescent Adherence Patient Tool.

^c^MDOT: mobile directly observed therapy.

Over a third of the references identified as eligible during title and abstract screening only had abstracts available (7/20, 35%) [31,35,36,39-42]. They were included in the analysis where relevant data were available; one of the abstracts only presented interim results [35]. Full texts were found for 3 of these 7 references [36,40,42], and data from those papers were used. A total of 4 studies were analyzed by 9 separate articles and abstracts: the ADolescent Adherence Patient Tool (ADAPT) study [25,33,34], a study comparing web-based Asthma Control Test and fractional exhaled nitric oxide monitoring with standard care [26,44], a study of inhaler electronic monitoring devices (EMDs) with audiovisual reminders [29,30], and a study of a real-time medication monitoring device with SMS text messaging reminders [31,43].

There was a wide range in study durations, from 3 weeks [32] to 24 months [27], with the most common length of follow-up being 6 or 12 months (n=4 [25,28-30,33,34,41] and n=3 studies [26,31,36,43,44] for each). There was also a wide range of numbers of participants included in the 15 studies, ranging from 22 [40] to almost 1200 [27], with an average of approximately 230 participants and a median of 209 [31,43].

There were no distinctive age categories that emerged from these studies. Of the 15 distinct studies, only 2 pairs used the same age range (4-11 years [31,38,43] and 6-16 years [36,42]). A total of 3 studies included adult participants, as well as child or adolescent participants [28,35,39]. The youngest participants included in the study were aged 3 years [27]. Of the studies that focused on participants under 18 years, the age range eligible for inclusion in each study ranged from 6 years (age 12-17 years [32]) to 15 years (age 4-18 years [26,44]).

A total of 4 studies took place across multiple centers [26,31,35,42-44], and most of the rest were associated with large medical centers [27,28,32,38] or clinics [36,41]. The remaining 5 studies were recruited from or associated with a hospital emergency department [29,30], community pharmacies [25,33,34], outpatient appointments in a hospital or Asthma Clinic [40], Howard University [39], and impoverished, rural school districts [37].

### Types of Digital Interventions

Various types of digital interventions for monitoring or improving medication adherence examined in the studies were included in this review (Table 1). The most common type of intervention, evaluated by a third of the studies (5/15, 33%), was EMDs. However, these EMDs varied in their features, which included audiovisual reminders [29,30], text messages [31,43], alarms [36], and app or web-based sources that could be synced to provide personal feedback [35,36], educational content [35], reminders [41], and capture adherence data [41].

Apps were another common intervention evaluated; 3 studies specifically evaluated three different app-based interventions. These included the ADAPT app that connects adolescents to their community pharmacist through a desktop application and enables them to monitor symptoms and adherence, chat with peers and their pharmacist, watch short educational movies, and set medication alarms [25,33,34]. Another app, CHANGE Asthma, was developed for children by 5 pediatricians and modified based on feedback from a pilot of 24 caregivers. It used short videos and games and an asthma action plan to improve asthma knowledge and control [38]. The third app evaluated (AsthmaWin) also included an asthma action plan but focused more on monitoring symptoms and medication adherence [39].

Other types of interventions evaluated included web-based monitoring programs [26,44] (one of which was a component of a Virtual Asthma Clinic [42]), a speech recognition automated telephone program to improve medication adherence [27], text message medication reminders [28], a website and text-based reminder system (MyMediHealth) [32], a remote directly observed therapy tool to improve inhaler use and adherence [40], and a school-based educational telemedicine intervention that provided interactive video sessions for children, caregivers, and school nurses [37].

### Evidence of Effectiveness

#### Overview

Several different outcome measures were used in the studies to evaluate the interventions, but the results regarding effectiveness were inconsistent. The outcome with the highest proportion of studies finding a significant, positive effect was for improving medication adherence. The reported effectiveness of interventions and improvement in asthma control and health outcomes were mixed. Patient feedback regarding acceptability and usability was generally high.

#### Treatment or Medication Adherence

A total of 10 studies evaluated the effectiveness of their interventions in improving treatment or medication adherence. Over two-thirds (7/10, 70%) reported significantly higher adherence in the intervention group compared with the control group [25,27,29,31,32,36,41,43]. Of the remaining 3 studies, one reported higher adherence in the intervention group compared with the control group, but no analysis of significance was provided [37], and one reported a trend toward improvement over time [40]. The final study, which evaluated an SMS text messaging reminder system, found a decline in adherence over the intervention and control periods in both groups [28].

Of the 7 studies that found a significant difference in adherence between groups, 4 were evaluating EMDs [29,31,41,43]. The others evaluated the speech recognition automated telephone program [27], the website and text-based reminder system (MyMediHealth) [32], and the ADAPT app [25].

Only one study each evaluated the effectiveness of improving inhaler use and symptom monitoring, both of which found improvements. Shields et al [40] found that remote directly observed therapy improved the inhaler technique equally in the immediate and delayed intervention groups. Perry et al [37] found significantly higher self-reports of peak flow meter use in the intervention group compared with the control group.

#### Asthma Control and Health Care Visits

There were very mixed results in 9 studies that evaluated asthma control as an outcome. Of the 9 studies, 4 found either no effect of the intervention on asthma control [25,35,43] or no significant difference between groups [38]. However, Real et al [38] found a significant positive association between the degree of app use and asthma control.

Another 4 studies reported improved asthma control in the intervention group compared with the control group [36,39,40,42], although only one of these studies demonstrated statistical significance [42]. Another one of these studies analyzed the 2 groups together and reported a significant improvement in asthma control over time [40]. The final study found that asthma control could be maintained after a clinically relevant reduction in inhaled corticosteroids in the web-based monitoring condition [26,44].

Only 2 studies evaluated the effect of the intervention on health care visits, but neither found any differences [27,42].

#### Health and Quality of Life Outcomes

The overall effect of the digital interventions on health outcomes remains unclear. Of the 9 studies that evaluated health outcomes (including quality of life or symptom-free days), 5 found no significant improvement [25,26,31,36,37,43,44]. However, 3 studies reported significant improvements in self-reported quality of life [32], asthma morbidity scores [29], and number of symptom-free days [42]. One study reported a significant improvement in parents’ self-reported quality of life over time and a nonsignificant trend toward improvement of the children’s quality of life [40].

#### Patient Perceptions, Acceptability, and Usability

A total of 6 studies examined outcomes related to patient perceptions, acceptability, or usability. These studies reported generally high satisfaction and acceptability [30,32-34], a desire to continue using the intervention [39,41], or positive feedback [40].

#### Cost-effectiveness

Only 1 study (2 articles) explicitly assessed cost-effectiveness [31,43]. The authors found that costs were higher in the intervention group, and although this difference was not statistically significant [43], the technology was deemed not cost-effective because it was not associated with significant improvements in health outcomes [31]. Upon closer inspection of reported mean adjusted costs per patient, although the hospital costs in the intervention arm are lower, the medication cost and parental production loss because of absence from paid work to care for children have been calculated to be higher by 16% and 141.8%, respectively [31]. Another study discussed the potential cost savings but did not analyze them as part of the study [36].

### Integration With Clinical Care Pathways

Half of the studies included in the review (8/15, 53% of studies or 10/20, 50% of articles) did not explicitly discuss how the digital intervention they were evaluating was integrated with clinical care pathways [28-32,38-41,43]. A few studies described sending data from the interventions back to physicians to update the patients’ health records or inform care, although this potential would likely be feasible for many of them. For the few that did, integration of the intervention with the health care system was generally reported positively.

Even among those that described a specific link between the intervention and the health care system, the specific details about integration were not the primary focus of the paper. For instance, one study noted that the intervention was built into routine clinical care in the study and described how data could be uploaded to a website for patients, parents or caregivers, and clinicians to review adherence data together at appointments [36]. Some of the studies that monitored symptoms or adherence produced treatment advice based on data analysis from the system algorithms [26,44] or sent physicians warnings if a patient was out of a certain threshold [35]. The Virtual Asthma Clinic, which also sent feedback to physicians if a patient’s asthma control scores were low, was found to be successful in increasing asthma control and symptom-free days and was proposed by the authors as a partial replacement for outpatient visits [42]. Details of how these systems were integrated with the health care system have not been described.

One study whose intervention was significantly integrated with the health care system was the ADAPT app study [25,33,34]. One of the aims of the intervention was to increase collaboration and communication between adolescents and pharmacists because of the increasing role of pharmacists as health care providers in the Netherlands [25]. Pharmacists involved in the intervention reported valuing the improved contact with patients and found the intervention satisfactory, useful in fulfilling their role, and not time-consuming [34]. This contrasted with the perceptions of pharmacists who did not participate in the intervention, who identified time constraints as a barrier to the use of mobile health [34]. However, a barrier was identified because the *stand-alone* desktop interface of the ADAPT app for pharmacists was not integrated with the pharmacy’s general information system [34]. This study highlights the potential value of deliberate and considers efforts to integrate new digital health technologies for asthma management with existing health systems.

Speech recognition telemedicine intervention was another study that demonstrated integration with the health care system, which was integrated with the hospital’s electronic health record (EpicCare) to provide personalized calls to patients and is compatible with all standard electronic health record systems [27].

The attempt of one study [37] to involve primary care providers in the intervention was not successful. Treatment prompts with medication recommendations based on caregiver reports and guidelines were provided to the participants’ primary care providers. These were found to be ineffective; of the 141 prompts sent out for individual participants, the request for feedback received a response from only 1 primary care provider [37].

## Discussion

### Principal Findings

Different varieties of studies were examined in this review; the study duration ranged from 3 weeks to 2 years, the number of participants ranged from 22 to 1187, and although the review was focused on children and adolescents, the range of ages studied was wide, with no distinct age groups emerging from the studies. There were also several different types of digital interventions analyzed in the RCTs, with EMDs and mobile apps being the most common. Moreover, the integration of these technologies with existing clinical care pathways and health systems has not been extensively discussed in most studies.

The review found inconsistent evidence for the effectiveness of digital technologies in achieving their various aims. Most support was found for the effectiveness of the interventions in improving treatment or medication adherence (7/10, 70% of studies found significant evidence of effectiveness). The results of studies assessing the impact of the intervention on asthma control and health outcomes were mixed, with some studies reporting positive effects and others showing no significant effect. Across the studies, evaluations of patient perceptions, acceptability, and usability were generally positive. Only one study evaluated the cost-effectiveness of these solutions, but because of insignificant improvement in health outcomes, the intervention was not found to be cost-effective [31].

### Limitations

One limitation of this review is that a risk of bias assessment was not performed on the studies. Although this is not a standard requirement for scoping reviews, it is a limitation of the study, as it would have contributed to the assessment of the first research question by providing an analysis of the quality of the research being conducted on technologically supported asthma pathways.

Another limitation is that the research questions and aims were adjusted after the search was performed. They were changed before any screening or selection took place but may have resulted in relevant articles being missed because the search terms were established for a slightly different scope. Owing to time limitations, no manual searches of the references of reviews retrieved in the initial search were performed, which could have resulted in eligible articles being overlooked.

### Meaning and Future Research

The large number of studies identified in the initial search and the variety of technological interventions to support pediatric asthma care demonstrate the broad scope of this research area. This review identified a few strong trends regarding how technologically supported asthma pathways for children and young people are being researched. The studies used a large range of sample sizes and participants of varying ages, which makes it difficult to make valid comparisons or conduct meta-analyses across different studies. A theoretical framework for determining what ages to study or how to stratify children and young people into age groups would be useful for the future. Currently, there is no consensus in the literature on how to group children of various ages for research, which is a significant limitation in the field.

This review found that there is a wide variety of digital interventions being explored. Although many of the studies examined reported positive results, strong evidence of their effectiveness in achieving various aims is still lacking. The strongest evidence was for improving treatment and medication adherence. However, the mixed evidence of asthma control, health, and quality of life outcomes suggests that there might be a disconnect between behavioral change and health outcomes. As asthma is a long-term condition, the study duration of included studies (from 3 weeks to 24 months) may not be long enough to observe significant health impacts, or there may be other factors influencing the relationship between treatment adherence and health outcomes (eg, technique). Understanding why this discrepancy was observed could help inform the design of more effective digital interventions and better study designs.

Another notable area that was missing from many of the studies was an assessment of the cost-effectiveness of the intervention. Considering that a key aim of many digital health technologies is to reduce the burden on health care systems by improving patient self-management, the benefit and cost of the intervention compared with the current standard of care is essential in the decision to integrate digital interventions into clinical care pathways. This will be a key area to consider for future evaluations of these technologies so that limited health care resources can be deployed to create the greatest value [45].

The overall findings are generally consistent with the previous reviews described in the *Introduction* section. Collectively, they identified at least some evidence of the benefits (depending on outcomes) of various digital health technologies on asthma-related outcomes [9-12,14]. One review also noted a lack of data regarding the cost-effectiveness of the digital asthma self-management interventions and patient perspectives [12]. This is also consistent with this review; patient perspectives were generally high when reported but were only examined in about a quarter (6/20, 30%) of the included studies.

Another key area for future research will be around the integration of these digital solutions into clinical pathways. As with cost-effectiveness, this review found that most studies did not explicitly consider or evaluate how the technology they were examining would interact with existing health systems. The potential benefit of integrating patient-reported data with patients’ health records to inform care plans and pathways is likely feasible for many of the technologies assessed but was not examined as a key outcome of the technology. Similarly, acceptability and usability data focused primarily on patient users. Understanding how these technologies can best support and interact with existing clinical pathways could help inform their design, improvement, and sustainable adoption.

### Conclusions

The purpose of this scoping review was to summarize the literature on technologically enhanced asthma care pathways for children and young people. A large body of research is ongoing in this area and spans a wide range of technologies and ages. Although there was some evidence for the effectiveness of the digital interventions examined, particularly for improving treatment and medication adherence, further research is needed to establish the effectiveness of the interventions in improving asthma control and other health outcomes. This apparent discrepancy between significant evidence for behavior change and a lack of significant evidence for subsequent health impacts should be further examined, as it could indicate factors other than treatment adherence that affect health outcomes and could also be targeted for intervention. A couple of gaps in the literature were identified in terms of cost-effectiveness and integration with existing care pathways. Both of these aspects are essential for the successful adoption, scale-up, and sustained use of digital health interventions and are key areas for future research.

## Appendix

Multimedia Appendix 1 PRISMA-ScR (Preferred Reporting Items for Systematic Reviews and Meta-Analyses extension for scoping reviews) checklist.

Multimedia Appendix 2 Search record.

Multimedia Appendix 3 EndNote search criteria.

Multimedia Appendix 4 Data extraction table.

## Abbreviations

ADAPT: ADolescent Adherence Patient Tool
CENTRAL: Cochrane Central Register of Controlled Trials
EMD: electronic monitoring device
MeSH: Medical Subject Headings
PRISMA-ScR: Preferred Reporting Items for Systematic Reviews and Meta-Analyses extension for scoping reviews
RCT: randomized controlled trial

## References

[1] Mallol J, Crane J, von Mutius E, Odhiambo J, Keil U, Stewart A, ISAAC Phase Three Study Group (2013). The International Study of Asthma and Allergies in Childhood (ISAAC) Phase Three: a global synthesis. Allergol Immunopathol (Madr).

[2] Lenney W, Bush A, Fitzgerald DA, Fletcher M, Ostrem A, Pedersen S, Szefler SJ, Zar HJ (2018). Improving the global diagnosis and management of asthma in children. Thorax.

[3] Childhood asthma. NHS England.

[4] (2015). Why asthma still kills National Review of Asthma Deaths. National Review of Asthma Deaths - Royal College of Physicians.

[5] Nunes C, Pereira AM, Morais-Almeida M (2017). Asthma costs and social impact. Asthma Res Pract.

[6] Wolfe I, Thompson M, Gill P, Tamburlini G, Blair M, van den Bruel A, Ehrich J, Pettoello-Mantovani M, Janson S, Karanikolos M, McKee M (2013). Health services for children in western Europe. Lancet.

[7] Lycett HJ, Raebel EM, Wildman EK, Guitart J, Kenny T, Sherlock J, Cooper V (2018). Theory-based digital interventions to improve asthma self-management outcomes: systematic review. J Med Internet Res.

[8] Tinschert P, Jakob R, Barata F, Kramer J, Kowatsch T (2017). The potential of mobile apps for improving asthma self-management: a review of publicly available and well-adopted asthma apps. JMIR Mhealth Uhealth.

[9] Unni E, Gabriel S, Ariely R (2018). A review of the use and effectiveness of digital health technologies in patients with asthma. Ann Allergy Asthma Immunol.

[10] Alquran A, Lambert KA, Farouque A, Holland A, Davies J, Lampugnani ER, Erbas B (2018). Smartphone applications for encouraging asthma self-management in adolescents: a systematic review. Int J Environ Res Public Health.

[11] Jeminiwa R, Hohmann L, Qian J, Garza K, Hansen R, Fox BI (2019). Impact of eHealth on medication adherence among patients with asthma: A systematic review and meta-analysis. Respir Med.

[12] Morrison D, Wyke S, Agur K, Cameron EJ, Docking RI, Mackenzie AM, McConnachie A, Raghuvir V, Thomson NC, Mair FS (2014). Digital asthma self-management interventions: a systematic review. J Med Internet Res.

[13] Stewart AC, Gannon KN, Beresford F, Fleming L (2018). Adolescent and caregivers' experiences of electronic adherence assessment in paediatric problematic severe asthma. J Child Health Care.

[14] Ramsey RR, Plevinsky JM, Kollin SR, Gibler RC, Guilbert TW, Hommel KA (2020). Systematic review of digital interventions for pediatric asthma management. J Allergy Clin Immunol Pract.

[15] Betz CL, Lewinter K, Kysh L, Hudson S, Espinoza J (2019). Smart devices for the management of pediatric asthma: a scoping review protocol. JBI Database System Rev Implement Rep.

[16] Steinhubl SR, Muse ED, Topol EJ (2015). The emerging field of mobile health. Sci Transl Med.

[17] Gaga M, Samitas K, Zervas E (2018). Inhaler adherence in severe asthma: is there an electronic solution?. Eur Respir J.

[18] Attaway AH, Alshabani K, Bender B, Hatipoğlu US (2020). The utility of electronic inhaler monitoring in COPD management: promises and challenges. Chest.

[19] (2019). Learn about the launch of the innovative smart inhaler device in Europe. Health Europa.

[20] Harris K, Grigg J (2019). The use of electronic monitoring devices in adherence with asthma medications, and their impact on patient outcomes: a systematic review PROSPERO registration. PROSPERO.

[21] Blakey JD, Bender BG, Dima AL, Weinman J, Safioti G, Costello RW (2018). Digital technologies and adherence in respiratory diseases: the road ahead. Eur Respir J.

[22] Schneider T, Panzera AD, Martinasek M, McDermott R, Couluris M, Lindenberger J, Bryant C (2016). Physicians' perceptions of mobile technology for enhancing asthma care for youth. J Child Health Care.

[23] Munn Z, Peters MD, Stern C, Tufanaru C, McArthur A, Aromataris E (2018). Systematic review or scoping review? Guidance for authors when choosing between a systematic or scoping review approach. BMC Med Res Methodol.

[24] Tricco AC, Lillie E, Zarin W, O'Brien KK, Colquhoun H, Levac D, Moher D, Peters MD, Horsley T, Weeks L, Hempel S, Akl EA, Chang C, McGowan J, Stewart L, Hartling L, Aldcroft A, Wilson MG, Garritty C, Lewin S, Godfrey CM, Macdonald MT, Langlois EV, Soares-Weiser K, Moriarty J, Clifford T, Tunçalp Ö, Straus SE (2018). PRISMA extension for Scoping Reviews (PRISMA-ScR): checklist and explanation. Ann Intern Med.

[25] Kosse RC, Bouvy ML, de Vries TW, Koster ES (2019). Effect of a mHealth intervention on adherence in adolescents with asthma: A randomized controlled trial. Respir Med.

[26] Beerthuizen T, Voorend-van Bergen S, van den Hout WB, Vaessen-Verberne AA, Brackel HJ, Landstra AM, van den Berg NJ, de Jongste JC, Merkus PJ, Pijnenburg MW, Sont JK (2016). Cost-effectiveness of FENO-based and web-based monitoring in paediatric asthma management: a randomised controlled trial. Thorax.

[27] Bender BG, Cvietusa PJ, Goodrich GK, Lowe R, Nuanes HA, Rand C, Shetterly S, Tacinas C, Vollmer WM, Wagner N, Wamboldt FS, Xu S, Magid DJ (2015). Pragmatic trial of health care technologies to improve adherence to pediatric asthma treatment: a randomized clinical trial. JAMA Pediatr.

[28] Britto MT, Rohan JM, Dodds CM, Byczkowski TL (2017). A randomized trial of user-controlled text messaging to improve asthma outcomes: a pilot study. Clin Pediatr (Phila).

[29] Chan AH, Stewart AW, Harrison J, Camargo CA, Black PN, Mitchell EA (2015). The effect of an electronic monitoring device with audiovisual reminder function on adherence to inhaled corticosteroids and school attendance in children with asthma: a randomised controlled trial. Lancet Respir Med.

[30] Chan AH, Stewart AW, Harrison J, Black PN, Mitchell EA, Foster JM (2017). Electronic adherence monitoring device performance and patient acceptability: a randomized control trial. Expert Rev Med Devices.

[31] Goossens LM, Vasbinder EC, Van den Bemt PM, Rutten-van Mölken MP (2014). Cost-effectiveness of real-time medication monitoring in children with asthma. Value Health.

[32] Johnson KB, Patterson BL, Ho Y, Chen Q, Nian H, Davison CL, Slagle J, Mulvaney SA (2016). The feasibility of text reminders to improve medication adherence in adolescents with asthma. J Am Med Inform Assoc.

[33] Kosse RC, Bouvy ML, Belitser SV, de Vries TW, van der Wal PS, Koster ES (2019). Effective engagement of adolescent asthma patients with mobile health-supporting medication adherence. JMIR Mhealth Uhealth.

[34] Kosse RC, Bouvy ML, de Vries TW, Koster ES (2019). Evaluation of a mobile health intervention to support asthma self-management and adherence in the pharmacy. Int J Clin Pharm.

[35] Merchant R, Inamdar R, Quade R, Van Sickle D, Maenner M, Patmas M (2013). Interim results from a randomized, controlled trial of remote monitoring of inhaled bronchodilator use on asthma control and management. Chest.

[36] Morton RW, Elphick HE, Rigby AS, Daw WJ, King DA, Smith LJ, Everard ML (2017). STAAR: a randomised controlled trial of electronic adherence monitoring with reminder alarms and feedback to improve clinical outcomes for children with asthma. Thorax.

[37] Perry TT, Halterman JS, Brown RH, Luo C, Randle SM, Hunter CR, Rettiganti M (2018). Results of an asthma education program delivered via telemedicine in rural schools. Ann Allergy Asthma Immunol.

[38] Real FJ, Beck AF, DeBlasio D, Zackoff M, Henize A, Xu Y, Davis D, Cruse B, Klein MD (2019). Dose Matters: A smartphone application to improve asthma control among patients at an urban pediatric primary care clinic. Games Health J.

[39] Reece ER, Burnette AF, Lewis-Land CJ (2017). Pilot study of Asthmawin mobile iphone app in the management of asthma. J Allerg Clin Immunol.

[40] Shields MD, ALQahtani F, Rivey MP, McElnay JC (2018). Mobile direct observation of therapy (MDOT) - A rapid systematic review and pilot study in children with asthma. PLoS One.

[41] Simoneau T, Sun Y, Gherlone N, Almeida S, Manice M, Hollenbach J (2019). A prospective, randomized, controlled study of inhaler electronic monitoring devices to improve adherence in children with asthma. Proceedings of the American Thoracic Society International Conference.

[42] van den Wijngaart LS, Roukema J, Boehmer AL, Brouwer ML, Hugen CA, Niers LE, Sprij AJ, Rikkers-Mutsaerts ER, Rottier BL, Donders AR, Verhaak CM, Pijnenburg MW, Merkus PJ (2017). A virtual asthma clinic for children: fewer routine outpatient visits, same asthma control. Eur Respir J.

[43] Vasbinder EC, Goossens LM, Rutten-van Mölken MP, de Winter BC, van Dijk L, Vulto AG, Blankman EI, Dahhan N, Veenstra-van Schie MT, Versteegh FG, Wolf BH, Janssens HM, van den Bemt PM (2016). e-Monitoring of Asthma Therapy to Improve Compliance in children (e-MATIC): a randomised controlled trial. Eur Respir J.

[44] Voorend-van Bergen S, Vaessen-Verberne AA, Brackel HJ, Landstra AM, van den Berg NJ, Hop WC, de Jongste JC, Merkus PJ, Pijnenburg MW (2015). Monitoring strategies in children with asthma: a randomised controlled trial. Thorax.

[45] Jamison D, Breman J, Measham A, Alleyne G, Claeson M, Evans D, Jha P, Mills A, Musgrove P (2006). Cost-effectiveness analysis. Priorities in Health.

