# Supplementary material for: Digital Technologies for Monitoring and Improving Treatment Adherence in Children and Adolescents With Asthma: Scoping Review of Randomized Controlled Trials
Source: JMIR Pediatr Parent. 2021 Sep 17;4(3):e27999. doi: 10.2196/27999 (PMC8486994; doi:10.2196/27999)
Supplement: Multimedia Appendix 2 [file pediatrics_v4i3e27999_app2.docx]

### Appendix B. Search record

| Database | Search String | Articles |
| --- | --- | --- |
| PubMed | ((asthma[MeSH Terms]) OR (Asthma[Title/Abstract] OR wheez*[Title/Abstract] OR dyspnea[Title/Abstract] OR cough*[Title/Abstract] OR (chest ADJ2 tight*)[Title/Abstract] OR “shortness of breath”[Title/Abstract])) AND ((Drug Therapy OR Medication Adherence OR Patient Compliance OR Treatment Adherence and Compliance OR Self-Management OR Disease Management OR Patient Education OR Patient Care Management[MeSH Terms]) OR (Self-management[Title/Abstract] OR “self care”[Title/Abstract] OR “self-care” [Title/Abstract] OR “disease management”[Title/Abstract] OR (medication[Title/Abstract] OR treatment[Title/Abstract] OR drug[Title/Abstract] OR patient ADJ3 adherence[Title/Abstract] OR compliance[Title/Abstract] OR persistence)[Title/Abstract] OR “patient education”[Title/Abstract] OR (treatment[Title/Abstract] OR care[Title/Abstract] OR action[Title/Abstract] OR asthma ADJ2 plan)[Title/Abstract] OR engagement[Title/Abstract] OR asthma control[Title/Abstract])) AND ((Adolescent OR Adolescent Health OR Child OR Child Health OR Pediatrics OR Family[MeSH Terms]) OR (Pediatric*[Title/Abstract] OR paediatric*[Title/Abstract] OR child[Title/Abstract] OR children[Title/Abstract] OR kid[Title/Abstract] OR kids[Title/Abstract] OR teen[Title/Abstract] OR teens[Title/Abstract] OR adolescen*[Title/Abstract] OR family[Title/Abstract] OR youth[Title/Abstract] OR “young people”[Title/Abstract] OR “young person”[Title/Abstract])) AND ((Cell Phone OR Telemedicine OR Computers OR Computers, Handheld OR Internet OR Internet-based Intervention OR Mobile Applications OR Internet of Things[MeSH Terms]) OR (“mHealth”[Title/Abstract] OR “mobile health”[Title/Abstract] OR “eHealth”[Title/Abstract] OR ((mobile[Title/Abstract] OR phone[Title/Abstract] OR smartphone[Title/Abstract] OR cell) ADJ3 “app” OR “apps” OR “application*”)[Title/Abstract] OR web[Title/Abstract] OR internet[Title/Abstract] OR online intervention[Title/Abstract] OR web-based intervention[Title/Abstract] OR digital intervention[Title/Abstract] OR virtual[Title/Abstract] OR web[Title/Abstract] OR “smart device*”[Title/Abstract] OR “IoT”[Title/Abstract] OR “internet of things”[Title/Abstract] OR “smart inhaler*”[Title/Abstract] OR monitor*[Title/Abstract] OR wearable[Title/Abstract])) | 2,858 |
| Cochrane Central Register of Controlled Trials (CENTRAL) | #1 MeSH descriptor: [Asthma] explode all trees  #2 MeSH descriptor: [Drug Therapy] explode all trees  #3 MeSH descriptor: [Patient Compliance] explode all trees  #4 (Asthma OR wheez* OR dyspnea OR cough* OR (chest  NEAR/2 tight*) OR “shortness of breath”):ti,ab,kw (Word  variations have been searched)  #5 MeSH descriptor: [Medication Adherence] explode all trees  #6 MeSH descriptor: [Treatment Adherence and Compliance]  explode all trees  #7 MeSH descriptor: [Self-Management] explode all trees  #8 MeSH descriptor: [Disease Management] explode all trees  #9 MeSH descriptor: [Patient Education as Topic] explode all  trees  #10 MeSH descriptor: [Patient Care Management] explode all  trees  #11 #2 OR #3 OR #5 OR #6 OR #7 OR #8 OR #9 OR #10  #12 MeSH descriptor: [Adolescent] explode all trees  #13 MeSH descriptor: [Adolescent Health] explode all trees  #14 MeSH descriptor: [Child] explode all trees  #15 MeSH descriptor: [Child Health] explode all trees  #16 MeSH descriptor: [Pediatrics] explode all trees  #17 MeSH descriptor: [Family] explode all trees  #18 #12 OR #13 OR #14 OR #15 OR #16 OR #17  #19 MeSH descriptor: [Cell Phone] explode all trees  #20 MeSH descriptor: [Telemedicine] explode all trees  #21 MeSH descriptor: [Computers] explode all trees  #22 MeSH descriptor: [Computers, Handheld] explode all trees  #23 MeSH descriptor: [Internet] explode all trees  #24 MeSH descriptor: [Internet-Based Intervention] explode all  trees  #25 MeSH descriptor: [Mobile Applications] explode all trees  #26 MeSH descriptor: [Internet of Things] explode all trees  #27 #19 OR #20 OR #21 OR #22 OR #23 OR #24 OR #25 OR  #26  #28 (Self-management OR “self care” OR “self-care” OR  “disease management” OR (medication OR treatment OR  drug OR patient NEAR/3 adherence OR compliance OR  persistence) OR “patient education” OR (treatment OR care  OR action OR asthma NEAR/2 plan) OR engagement OR  asthma control):ti,ab,kw  #29 (Pediatric* OR paediatric* OR child OR children OR kid  OR kids OR teen OR teens OR adolescen* OR family OR  youth OR “young people” OR “young person”):ti,ab,kw  #30 (“mHealth” OR “mobile health” OR “eHealth” OR ((mobile  OR phone OR smartphone OR cell) NEAR/3 "app" OR  "apps" OR "application*") OR web OR internet OR online  intervention OR web-based intervention OR digital  intervention OR virtual OR web OR “smart device*” OR  “IoT” OR “internet of things” OR “smart inhaler*” OR  monitor* OR wearable):ti,ab,kw  #31 #1 OR #4  #32 #11 OR #28  #33 #18 OR #29  #34 #27 OR #30  #35 #31 AND #32 AND #33 AND #34 with Publication Year  from 2014 to 2020, in Trials | 755 |
| Web of Science | TS=(Asthma OR wheez* OR dyspnea OR cough* OR (chest NEAR/2 tight*) OR “shortness of breath”) AND TS=(Drug Therapy OR Medication Adherence OR Patient Compliance OR Treatment Adherence and Compliance OR Patient Care Management OR Self-management OR “self care” OR “self-care” OR “disease management” OR (medication OR treatment OR drug OR patient NEAR/3 adherence OR compliance OR persistence) OR “patient education” OR (treatment OR care OR action OR asthma NEAR/2 plan) OR engagement OR asthma control) AND TS=(Adolescent OR Adolescent Health OR Child OR Child Health OR Pediatrics OR Family OR Pediatric* OR paediatric* OR children OR kid OR kids OR teen OR teens OR adolescen* OR youth OR “young people” OR “young person”) AND TS=(Cell Phone OR Telemedicine OR Computers OR Computers, Handheld OR Internet OR Internet-based Intervention OR Mobile Applications OR “mHealth” OR “mobile health” OR “eHealth” OR ((mobile OR phone OR smartphone OR cell) NEAR/3 app*) OR web OR online intervention OR web-based intervention OR digital intervention OR virtual OR “smart device*” OR “IoT” OR “internet of things” OR “smart inhaler*” OR monitor* OR wearable) | 1,505 |
| EMBASE (Ovid) | (asthma/ or (asthma or wheez* or dyspnea or cough* or (chest adj2 tight*) or shortness of breath).ti,ab.) AND (drug therapy/ or medication compliance/ or patient compliance/ or self care/ or disease management/ or patient education/ or (Self-management or self care or self-care or disease management or (((medication or treatment or drug or patient) adj3 adherence) or compliance or persistence) or patient education or ((treatment or care or action or asthma) adj2 plan) or engagement or asthma control).ti,ab.) AND (adolescent/ or adolescent health/ or child/ or child health/ or pediatrics/ or family/ or (Pediatric* or paediatric* or child or children or kid or kids or teen or teens or adolescen* or family or youth or young people or young person).ti,ab.) AND (mobile phone/ or telemedicine/ or computer/ or personal digital assistant/ or Internet/ or web-based intervention/ or mobile application/ or “internet of things”/ or (mHealth or mobile health or eHealth or ((mobile or phone or smartphone or cell) adj3 app*) or web or internet or online intervention or web-based intervention or digital intervention or virtual or web or smart device* or IoT or internet of things or smart inhaler* or monitor* or wearable).ti,ab.) | 1,098 |
| PsycINFO (ProQuest) | ((ab(Asthma OR wheez* OR dyspnea OR cough* OR (chest NEAR/2 tight*) OR "shortness of breath")) OR (ti(Asthma OR wheez* OR dyspnea OR cough* OR (chest NEAR/2 tight*) OR "shortness of breath"))) AND ((ab(Drug Therapy OR Medication Adherence OR Patient Compliance OR (Treatment NEAR/2 Adherence OR Compliance) OR Patient Care Management OR Self-management OR "self care" OR "self-care" OR "disease management" OR (medication OR treatment OR drug OR patient NEAR/3 adherence OR compliance OR persistence) OR "patient education" OR (treatment OR care OR action OR asthma NEAR/2 plan) OR engagement OR asthma control)) OR (ti(Drug Therapy OR Medication Adherence OR Patient Compliance OR (Treatment NEAR/2 Adherence OR Compliance) OR Patient Care Management OR Self-management OR "self care" OR "self-care" OR "disease management" OR (medication OR treatment OR drug OR patient NEAR/3 adherence OR compliance OR persistence) OR "patient education" OR (treatment OR care OR action OR asthma NEAR/2 plan) OR engagement OR asthma control))) AND ((ab(Adolescent OR Adolescent Health OR Child OR Child Health OR Pediatrics OR Family OR Pediatric* OR paediatric* OR children OR kid OR kids OR teen OR teens OR adolescen* OR youth OR "young people" OR "young person")) OR (ti(Adolescent OR Adolescent Health OR Child OR Child Health OR Pediatrics OR Family OR Pediatric* OR paediatric* OR children OR kid OR kids OR teen OR teens OR adolescen* OR youth OR "young people" OR "young person"))) AND ((ab(Cell Phone OR Telemedicine OR Computers OR (Computer* NEAR/1 Handheld) OR Internet OR Internet-based Intervention OR Mobile Applications OR "mHealth" OR "mobile health" OR "eHealth" OR ((mobile OR phone OR smartphone OR cell) NEAR/3 “app” OR “apps” OR “application*”) OR web OR online intervention OR web-based intervention OR digital intervention OR virtual OR "smart device*" OR "IoT" OR "internet of things" OR "smart inhaler*" OR monitor* OR wearable)) OR (ti(Cell Phone OR Telemedicine OR Computers OR (Computer* NEAR/1 Handheld) OR Internet OR Internet-based Intervention OR Mobile Applications OR "mHealth" OR "mobile health" OR "eHealth" OR ((mobile OR phone OR smartphone OR cell) NEAR/3 “app” OR “apps” OR “application*”) OR web OR online intervention OR web-based intervention OR digital intervention OR virtual OR "smart device*" OR "IoT" OR "internet of things" OR "smart inhaler*" OR monitor* OR wearable))) | 98 |

^a^Web of Science and PsycINFO do not have a specific search for MeSH terms, so all keywords and MeSH terms were included (with exact duplicates removed). In Web of Science, they were searched for in ‘Topic’, which searches title, abstract, author keywords, and Keywords Plus. In PsycINFO, they were searched for in Title and Abstract.
