# Supplementary material for: Digital Technologies for Monitoring and Improving Treatment Adherence in Children and Adolescents With Asthma: Scoping Review of Randomized Controlled Trials
Source: JMIR Pediatr Parent. 2021 Sep 17;4(3):e27999. doi: 10.2196/27999 (PMC8486994; doi:10.2196/27999)
Supplement: Multimedia Appendix 3 [file pediatrics_v4i3e27999_app3.docx]

### Appendix C: Endnote search criteria

| **Pass^a^** | **Search string** | **# of references remaining** |
| --- | --- | --- |
| 1 | Year = greater than or equal to 2014 | 5242 |
| 2 | Title = NOT (review OR protocol OR case OR guideline* OR handbook* OR position paper OR meta-analysis OR conference OR congress) | 4402 |
| 3^b^ | Any Field = Cell Phone OR smartphone OR mobile OR telemedicine OR Internet OR web OR online OR digital OR virtual OR mHealth OR eHealth | 1691 |
| 4^b^ | Any Field = smartphone app* OR phone app* OR mobile app* OR smart device* OR smart inhaler* OR internet of things OR IoT OR wearable OR Title = monitor* | 469 |
| 5 | Pass 3 AND 4 (with duplicates removed) | 1892 |
| 6 | Any Field = asthma NOT (dermatitis OR food allerg* OR sickle cell OR cystic OR cancer OR carcinoma OR diabetes OR pregnan* OR bowel OR virus OR viral) | 1119 |
| 7 | Any Field = NOT (biomarker* OR phenotype* OR genotype* OR genetic* OR agonist* OR enzyme) | 966 |
| 8 | Any Field = NOT (anaphylaxis OR immunotherapy OR influenza OR flu OR vaccine*) | 839 |
| 9 | Title = NOT (adult* OR face mask* OR access OR design OR method OR oscillometry OR FENO OR fractional exhaled nitric oxide OR asthma control assessment) | 740 |
| 10 | Any Field = child* OR teen* OR adolescen* OR youth* OR family OR parent* OR caregiver* OR paediatric* OR pediatric* | 702 |
| 11 | Any Field = NOT (oximetry OR spirometry OR physiotherap* OR phototherap* OR intubation OR injection OR expiratory variability OR breath temperature OR lung sound) | 626 |
| 12 | Any Field = NOT (optic OR ADHD OR eczema OR cardi* OR metabolic OR otitis OR sleep OR diet OR drug misuse OR withdrawal) | 544 |
| 13 | Any Field = NOT (antileukotriene OR inflammatory marker* OR omalzimub OR eosinophil* OR tiotropium), Title = NOT (college OR university OR military OR drug misuse) | 528 |
| 14 | Any Field = NOT (oscillation OR motivational interview* OR body weight OR hair OR claims data OR wikipedia OR environmental factor* OR nutrition OR RSV) | 495 |
| 15 | Any Field = NOT (microbiome OR pathogen* OR bronchiectasis OR vascular disease OR dysplasia OR infection OR tuberculosis OR toxocara OR depression) | 471 |
| 16 | Any Field = NOT (gastro* OR multisystemic OR transport OR forum OR antibiotic OR inpatient OR PM2.5 OR acupuncture OR administration form) | 436 |
| 17 | Any Field = develop* OR creat* NOT (evaluat* OR accept* OR feasibility OR assess* OR pilot OR usability OR test*) | 408 |
| 18 | Any Field = adhere* OR monitor* | 262 |
| 19 | Any Field = randomised control* OR randomized control* | 92 |

^a^Each pass was conducted on the subset of studies retrieved in the previous pass.

^b^EndNote was unable to include all of the search terms at once, so passes 3 and 4 were both conducted on pass 2 and then combined (with duplicates removed) in pass 5
