# Supplementary material for: Digital Technologies for Monitoring and Improving Treatment Adherence in Children and Adolescents With Asthma: Scoping Review of Randomized Controlled Trials
Source: JMIR Pediatr Parent. 2021 Sep 17;4(3):e27999. doi: 10.2196/27999 (PMC8486994; doi:10.2196/27999)
Supplement: Multimedia Appendix 4 [file pediatrics_v4i3e27999_app4.docx]

### Appendix D. Data Extraction Table

| Paper | RQ1. How is research on tech-supported asthma pathways being designed and conducted? | RQ 2. What is known about the effectiveness of tech to support treatment adherence and remote symptom monitoring? | RQ 3. What is the state of knowledge about integrating tech into clinical care pathways? |
| --- | --- | --- | --- |
| [35]  NOTE: same study as Voorend-van Bergen | - 1 year study, children 4-18, comparing monitoring strategies to adapt care - standard care (ACT once every 4 months) vs monthly web-ACT vs ACT every 4 months + FENO  - Recruited by their own paediatrician from general hospitals and tertiary referral centres  - Multicentre, prospective, partly blinded, parallel-group, three-arm randomised controlled superiority trial on monitoring strategies in asthmatic children with a follow-up of 1 year | - neither strategy improved symptom free days compared to standard care after 1 year  - Monthly web-based ACTs resulted in a clinically relevant decrease of ICS dose, while maintaining asthma control.  - FENO group: increase in asthma control in children under 12 | -not specifically discussed  - both strategies provided in addition to usual care - integrated? |
| [41] | -24 month study, ages 3-12, computerized speech recognition telephone intervention vs usual care condition  - SR program created with parent focus groups, script development, and beta testing  - personalised with data from EHR and automated, option to get callback from nurse/pharmacist  - study was conducted within Kaiser Permanente Colorado, a large, group-model health maintenance organization | -inhaled corticosteroid adherence was 25.4% higher in the intervention group than in the usual care group (24-month mean [SE] adherence, 44.5% [1.2%] vs 35.5% [1.1%], respectively; P < .001)  - no differences in healthcare visits  - strong potential for low-cost SR adherence programs integrated with an electronic health record | - intervention integrated in addition to usual care  - integrated with an electronic health record  - more than half of physicians use an EHR and current projections indicate that more than 90% of clinicians will be using an EHR within the decade.^21^ Further, the intervention in this study leveraged EpicCare, the most widely used ambulatory care EHR in the United States. The capacity to identify and sort patients by EHR indicators in this project is standard in all EHR systems, including eClinicalWorks, McKesson, Cerner, Allscripts, and GE Healthcare |
| [42] | -64 patients age 12 to 22 years with a diagnosis of poorly controlled persistent asthma in a 6-month longitudinal crossover study  - Adherence was objectively monitored in 22 of the participants  - participants were given the opportunity to personalize their text message reminders. They could schedule non–asthma medication reminders, appointment reminders, or other messages of their choice | - adherence declined in both groups over the intervention and control periods. This suggests that 3 months of messages did not create a long-term habit of ICS use | - not discussed explicitly  - were recruited at clinic visits  - should be easy to incorporate alongside standard care |
| [37] | - patients aged 6–15 years who attended the regional emergency department  - followed up every 2 months for 6 months  - randomly assigned patients to receive an electronic monitoring device for use with their preventer inhaler with the audiovisual reminder functions either enabled to support adherence to inhaled corticosteroids (intervention group) or disabled (control group) | - Median percentage adherence was 84% (10th percentile 54%, 90th percentile 96%) in the intervention group, compared with 30% (8%, 68%) in the control group (p<0·0001)  - significant improvements in adherence to inhaled corticosteroids in school-aged children with asthma  - change in asthma morbidity score from baseline to 6 months was significantly greater in the intervention group than in the control group (p=0·008) (more reduce in int group) | - Not explicitly discussed |
| [38] | - Children 6 to 15 years presenting with asthma to the hospital emergency department and prescribed inhaled corticosteroids were included  - Device quality control tests were conducted. Questionnaires on device acceptability, utility and ergonomics were completed at six months | - Acceptability scores were high, with higher scores in the reminder than non-reminder group (median, 5th-95th percentile: 4.1, 3.1–5.0 versus 3.7, 2.3–4.8; p < 0.001).  - Most (>90%) rated the device easy to use.  - Feedback was positive across five themes: device acceptability, ringtone acceptability, suggestions for improvement, effect on medication use, and effect on asthma control  - failure rates of 13–16% indicate the importance of quality control | - Not specifically discussed |
| [28] *NOTE: conference abstract only | - multi-center, randomized controlled trial. Included were 209 children (<12 years) with moderate to severe asthma, followed for 12 months  - all received a Real-Time Medication Monitoring system, in intervention group it sent texts to parents whose child appeared to miss a dose | - RTMM increases inhalation adherence, but there is no evidence of better health outcomes in this patient population within the first year. In these circumstances, this is not a cost-effective intervention.  - Costs were higher in the intervention group | - Not specifically discussed |
| [40] | - impact of MyMediHealth (MMH) – a website and a short messaging service (SMS)-based reminder system – on medication adherence and perceived self-efficacy in adolescents with asthma  - block-randomized controlled study in academic pediatric outpatient settings, 98 adolescents enrolled  - MMH users were asked to create a medication schedule and receive SMS reminders at designated medication administration times for 3 weeks | - Compared to controls, we found improvements in self-reported medication adherence (P = .011), quality of life (P = .037), and self-efficacy (P = .016).  - Subjects reported high satisfaction with MMH; however, the level of system usage varied widely  - significant racial disparity in the rate of MMH adoption | - not specifically discussed |
| [25] | - Cluster randomized controlled trial in 66 Dutch community pharmacies.  - Asthma patients aged 12–18 years were invited to participate, based on pharmacy medication refill records.  - 234 adolescents (147 in the control group and 87 in the intervention group) completed the study  - The main study outcome was self-reported medication adherence, measured with the Medication Adherence Report Scale (MARS).  - Secondary outcomes were asthma control and quality of life. Outcomes were measured at start (t = 0 months) and at the end of follow-up (t = 6 months) | - We did not find an intervention effect in the total study population, i.e., only in non-adherent patients.  - there was a positive effect of the intervention on medication adherence (MARS +2.12, p = 0.04).  - This effect was stronger (MARS +2.52, p = 0.02) in poor adherent adolescents with uncontrolled asthma (n = 74).  - No effect of the intervention was observed on asthma control or quality of life. | - current study used pharmacists as the healthcare provider, because pharmacists are increasingly expected to support appropriate use of medication in integrated care settings  - Increased collaborations between pharmacists and physicians may facilitate the identification of uncontrolled patients with low adherence rates.  - Pharmacists can subsequently support these patients with their medication use, by implementing mHealth interventions.  - Adherence could also be measured by using pharmacy refill records. However our study period covered only six months, and in the Netherlands patients usually collect chronic medication once every three months. |
| [34] *NOTE: same study as above | - explore experiences, barriers, and facilitators of pharmacists and patients towards the use of the interactive ADolescent Adherence Patient Tool (ADAPT)  - the perceptions of pharmacists towards mHealth interventions in general were explored  - setting: dutch community pharmacies | - Most patients (78%) would recommend the ADAPT intervention to others, and thought that the pharmacy was the right place for mHealth aiming to support adherence (63%).  - The possibility to monitor asthma symptoms was highly appreciated by patients and pharmacists.  - Pharmacists were satisfied with ADAPT intervention (96%), and using the intervention was not time consuming (91%).  - The ADAPT intervention promoted contact with patients (74%) and facilitated the healthcare providing role of pharmacists (83%). | - Providing extra care for patients was one of the main reasons for using mHealth (by both pharmacist groups).  - Pharmacists who delivered the ADAPT intervention valued the improved patient contact  - Another important facilitator for further implementation is the integration of mHealth in the pharmacy information system, because a ‘stand-alone’ desktop program restrained the integration with the pharmacist’s workflow  - MHealth interventions can facilitate the pharmacist’s responsibilities and promote contact with patients. This is important nowadays, because pharmacists are expected to combine their management role with more healthcare providing roles, and there is an ongoing shift towards integrated care settings |
| [33] *NOTE: same study as above | - Explore the use and the effective engagement of adolescents (aged 12 to 18 years) with the Adolescent Adherence Patient Tool (ADAPT)  - The ADAPT app was connected to a desktop application of the patient’s own community pharmacist  - included: questionnaire to monitor symptoms, peer and pharmacist chats, medication alarm, short movies, adherence questions | - 86 adolescents (mean age 15.0, SD 2.0 years) used the ADAPT app 17 times (range 1-113) per person. Females used the app more often than males (P=.01) and for a longer period of time (P=.03).  - The questionnaires to monitor symptoms and adherence were used by most adolescents.  - The total app use did not affect adherence; however, activity in the pharmacist chat positively affected medication adherence (P=.03), in particular, if patients sent messages to their pharmacist (P=.01)  - Adolescents have different preferences when using an mHealth app, as there was a wide variety in app usage per person  - The questionnaires to monitor asthma symptoms and adherence were used by most adolescents, which provided valuable data for health care providers and patients. Moreover, the use of the pharmacist chat positively affected adherence. |  |
| [26] *NOTE: abstract only, interim results | - intervention: inhaler sensors, personalised feedback and educational content (apps and online interfaces)  - control group had sensors only  - children and adults (5-80)  - 490 enrolled, 368 active/completed at 16 months | - sig diff in rescue inhaler use between groups after 100 days (fewer for int group)  - | - physicians could monitor intervention group patients and received proactive warnings if they fall below a threshold |
| [27] *NOTE: abstract only, interim results | - complex intervention comprising electronic adherence monitoring with feedback and reminder alarms can improve clinical outcomes and adherence in childhood asthma  - 90 participants were recruited, 25 have completed study, and 60% of follow-up visits have been completed  - Children with asthma in the UK were recruited and followed up in standard clinics for 12 months | - At 12 months, the mean adherence in the intervention arm is currently 83%, compared to 34% in the control arm  - The mean number of oral steroid courses in the 12 months is 1.7 in the intervention group and 2.7 in the control group | - not specifically discussed |
| [44] | - cluster randomized trial with rural children, ages 7–14 years (393 enrolled), comparing a school-based telemedicine asthma education intervention to usual care  - Each student participated in five 30–45 minute age-appropriate asthma education sessions via telemedicine (live interactive video)  - Telemedicine sessions were also conducted for intervention parents/caregivers and school nurses. Each 60–90 minute session was conducted at the school (local community center on weekend/nights). Caregivers participated in 2 sessions and school nurses participated in 1 session.  - Telemonitoring sessions were completed prior to the first child education session and at 3 months. During the session, intervention participants described symptoms for the preceding 2 weeks and completed the PedsQL 3.0 survey | - At the end of the intervention, there were no statistically significant differences in reported symptom free days (primary outcome) for either the intervention or usual care group.  - Participants in the intervention group reported significantly higher utilization of peak flow meters to monitor asthma and reported taking their asthma medications as prescribed more frequently when compared to the usual care group.  - There were no changes in other outcome measures including quality of life, self-efficacy, asthma knowledge, or lung function between groups  - Although there was some evidence of behavior change among intervention participants, these changes were inadequate to overcome the significant morbidity experienced by this highly symptomatic rural, impoverished population. | - The Primary Care Provider of each intervention participant received a prompt with guidelines-based asthma management19 at baseline and 3 months. Caregivers and school nurses received copies of the prompt to reinforce recommendations. The prompt included: 1) a summary of education sessions, 2) blank AAP with completion instructions, 3) synopsis of caregiver-reported symptoms and medications and 4) treatment recommendations according to guidelines.19 For example, if a participant’s caregiver reported uncontrolled, recommendations for initiation or step-up of controller therapy were given.19 PCPs received a 7-question survey to confirm receipt of the prompt, verify accuracy of asthma severity and control assessment. We provided a self-addressed stamped envelope to return the survey and phone/fax/mailing contacts for the research team to answer any questions or to receive additional feedback.  - Simply providing a treatment prompt to PCPs with medication recommendations was proven to be ineffective. |
| [43] | - developed and implemented a smartphone application (app) leveraging gamified features entitled CHANGE Asthma (“Clinic, Home, And on the Go Education for Asthma”). We subsequently assessed its impact on asthma control.  - Patients aged 4–11 years with a previously documented childhood asthma control test (C-ACT) score of <20, indicating poor control, were recruited  - App usage was monitored for 4 months  - curriculum iteratively developed by 5 pediatricians and 2 app developers  - piloted with caregivers and modified accordingly | - The control and intervention groups both included 20 caregivers with 75% of participants completing follow-up.  - Although C-ACT scores among intervention participants significantly improved at follow-up, compared to their own baseline (P = 0.04), the change of C-ACT score did not significantly differ from that of the control group (P = 0.78).  - Among the intervention participants, there was a positive, dose-dependent relationship between app usage time and positive change in C-ACT score (P = 0.03). | - not discussed |
| [29] *NOTE: abstract only | - AsthmaWin is an iPhone app, developed by CooperSoft Inc. that incorporates a physician-generated asthma action plan & involves daily recording of: (a) peak flow measurements, (b) medication usage—documented with a self-photo, (c) daily symptoms with an automatic reminder to take medications and that can reward completion of data, is intended to enhance patient physician communication  - whether the use of this app would improve medication adherence & improve asthma self-management as measured by Asthma Control Test (ACT) scores and peak expiratory flow rate (PEFR) measurements  - Forty eight asthmatics, predominantly African American, 13-60 years old were recruited from the Howard University Faculty Practice Plan  - | - Documented daily use of the Asthma Action plan leads to improvement in ACTs. PEFRs and medication adherence. Focus groups indicated patient willingness to use the app on an ongoing basis to assist in asthma management and an unwillingness to continue paper journaling.  - Reported controller medication usage was 86% among app users and 90% in the paper group. ACT scores in the app group improved by a mean of 3.8 points and by 2.4 points in the paper group. PEFRs improved an average of 9.09% in the app group and 7.82% in the paper group. | - not discussed, although enhanced communication was an aim |
| [30]*NOTE: abstract only | - determine if remote directly observed therapy (R-DOT) could monitor adherence and inhaler technique in children with Difficult to Treat Asthma (DTA)  - pilot study: 22 children (aged <15 yrs) with DTA were randomised to either immediate R-DOT for 6 weeks or delayed entry (R-DOT after 6 weeks) and asthma control was assessed at 12 weeks.  - mobile phone platform was used to record and send a short video clip (timed and dated, twice a day) of children using their inhaler. The videos were reviewed by a nurse and if needed the child/parent was provided with reminders/re-instruction | - Despite all children being able to demonstrate good inhaler technique at study entry, 80% were deemed not to have good inhaler technique while using R-DOT at home during the first week.  - By the end of the 3rd week after nurse led re-instructions all children had good inhaler technique. Both groups improved equally.  - R-DOT can test for and improve inhaler technique and monitor adherence and can be used to ensure that a period of optimised therapy has been delivered in DTA. The approach is not device specific and can be used with any type of inhaled therapy.  - Engagement with the process was generally good although cases of non-adherence with video uploads included living with a different parent at weekends, life too busy and mobile phone memory issues | - not discussed in abstract, but intervention required nurse involvement |
| [31] *NOTE: abstract only | - 8-17 years, 43 enrolled  - Breathe Smart EMD, one each for rescue and controller inhalers  - synced with mobile app, sends reminders and captures adherence data | - adherence based on pharmacy records after 3 months was significantly greater in int group than control, equal to mean adherence captured by EMD  - 83% desired to continue using EMD  - however, adherence in both groups still low | - not discussed |
| [32] *Abstract only | - multicentre, randomised controlled trial with a 16-month follow-up, 210 asthmatic children (6–16 years) treated in eight Dutch hospitals were randomised to usual care (4-monthly outpatient visits) and online care using a virtual asthma clinic (VAC) (8-monthly outpatient visits with monthly web-based monitoring)  - In between VAC visits, asthma control was monitored online, filling in a (C-)ACT monthly.  - If the (C-)ACT score was ≥20, automatic default messages were emailed with positive and encouraging content.  - If the (C-)ACT score was <20, feedback to the participants included advice to check their medication use, an individual action plan and a request to contact their asthma team when symptoms persisted.  - In addition, feedback was sent to the asthma team with the request to contact the participant within 2 working days to address the clinical condition of that moment. | - After follow-up, symptom-free days differed statistically between the usual care and VAC groups (difference of 1.23 days, 95% CI 0.42–2.04; p=0.003) in favour of the VAC. In terms of asthma control, the Childhood Asthma Control Test improved more in the VAC group (difference of 1.17 points, 95% CI 0.09–2.25; p=0.03). No differences were found for other outcome measures. | - Routine outpatient visits can partly be replaced by monitoring asthmatic children via eHealth.  - physicians contacted if issues identified  - demonstrates that eHealth using the VAC can substitute 50% of routine outpatient visits in paediatric asthma care while the number of SFDs improved significantly after a 16-month follow-up |
| [39] *NOTE same as Goosens study | - multicentre, randomised controlled trial, 209 children (aged 4–11 years) using ICS were recruited from five outpatient clinics and were given an real-time medication monitoring (RTMM) device for 12 months  - The intervention group also received tailored SMS reminders, sent only when a dose was at risk of omission | - Mean adherence was higher in the intervention group: 69.3% versus 57.3% (difference 12.0%, 95% CI 6.7%–17.7%).  - No differences were found for asthma control, quality of life or asthma exacerbations.  - Costs were higher in the intervention group, but this difference was not statistically significant | - not discussed |
| [36] | - multicentre trial with a 1-year follow-up, children aged 4–18 years with a doctor's diagnosis of asthma treated in seven hospitals were randomised to one of the three groups. In the web group, treatment was adapted according to ACT obtained via a website at 1-month intervals; in the FENO group according to ACT and FENO, and in the SC group according to the ACT at 4-monthly visits  - 280 included, 268 completed | - change from baseline in symptom-free days did not differ between monitoring strategies. With web-based ACT monitoring, ICS could be reduced substantially while control was maintained. | - treatment was adapted monthly according to the web-based ACT score, while in the FENO group, treatment was adapted to FENO and ACT score at clinic visits every 4 months  - The treating physicians were blinded to randomisation group, FENO and ACT. The local investigators, unblinded to ACT and FENO, provided the physicians with treatment advice based on the study algorithms and on the treatment plan  - Physicians could deviate from this advice for documented clinical reasons only. |
